# 100% selective cyclotrimerization of acetylene to benzene on Ag(111)†

**DOI:** 10.1039/d4sc01053a

**Published:** 2024-04-10

**Authors:** Volkan Çınar, Shengjie Zhang, Elizabeth E. Happel, Nipun T. S. K. Dewage, Matthew M. Montemore, E. Charles H. Sykes

**Affiliations:** a Department of Chemistry, Tufts University Medford Massachusetts 02155 USA charles.sykes@tufts.edu; b Department of Chemical and Biomolecular Engineering, Tulane University New Orleans Louisiana 70118 USA mmontemore@tulane.edu

## Abstract

Benzene, a high-volume chemical, is produced from larger molecules by inefficient and environmentally harmful processes. Recent changes in hydrocarbon feedstocks from oil to gas motivate research into small molecule upgrading. For example, the cyclotrimerization of acetylene reaction has been demonstrated on Pd, Pd alloy, and Cu surfaces and catalysts, but they are not 100% selective to benzene. We discovered that acetylene can be converted to benzene with 100% selectivity on the Ag(111) surface. Our temperature programmed desorption experiments reveal a threshold acetylene surface coverage of ∼one monolayer, above which benzene is formed. Furthermore, additional layers of acetylene increase the amount of benzene produced while retaining 100% selectivity. Our scanning tunneling microscopy images show that acetylene prefers square packing on the Ag(111) surface at low coverages, which converts to hexagonal packing when acetylene multilayers are present. Within this denser layer, features consistent with the proposed C_4_ intermediates of the cyclotrimerization process are observed. Density functional theory calculations demonstrate that the barrier for forming the crucial C_4_ intermediate generally decreases as acetylene multilayers are formed because the multilayer interacts more strongly with the surface in the transition state than in the initial state. Given that acetylene desorbs from Ag(111) at ∼90 K, the C_4_ intermediate on the pathway to benzene must be formed below this temperature, implying that if Ag-based heterogeneous catalysts can be run at sufficiently high pressure and low enough temperature, efficient and selective trimerization of acetylene to benzene may be possible.

## Abstract 2

If a critical acetylene surface coverage threshold above one monolayer is attained, acetylene conversion to benzene occurs with 100% selectivity on Ag(111). The presence of multiple monolayers has the unforeseen effect of increasing benzene production while maintaining selectivity.

## Graphical abstract

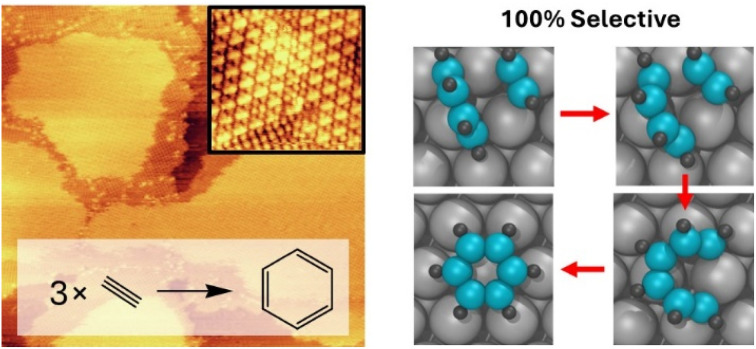

## Introduction

Benzene is a key compound in the chemical industry with a market value of ∼$40 billion in 2022 which is projected to reach ∼$80 billion by 2030.^1,2^ Benzene is widely used as a chemical intermediate for the manufacturing of various consumer goods such as cleaning products, clothing, packaging, building materials, and pharmaceuticals.^3^ Currently, benzene is mainly produced *via* three different methods which rely heavily on petroleum and coal, namely, catalytic reforming, hydrodealkylation of toluene, and steam cracking, each of which contribute ∼30% to the market supply.^4^ These processes form benzene from >C_6_ molecules and only the hydrodealkylation of toluene provides high conversion (∼90%) and selectivity (∼95%).^5^ The high cost and environmental impact of current benzene production methods – including severe operating conditions, high energy demand, and environmental pollution – incentivize the search for alternative routes.^6,7^ Furthermore, the recent surge in shale gas production in the United States has created a plentiful supply of C_1,2,3_ species; therefore, identification of methods for converting these light hydrocarbons into value added C_6+_ products would be timely.^8^ Thus, acetylene trimerization to benzene may be attractive in its own right, and is also representative of a class of C–C coupling reactions that are of increasing technological interest.

Previous work has shown that it is possible to produce benzene from acetylene on a variety of metal surfaces under both ultra-high vacuum (UHV) and reaction conditions.^9–35^ For example, Tysoe *et al.* showed acetylene forms benzene and ethylene on a Pd(111) surface and that increasing the surface acetylene coverage increased the yield of benzene.^36^ In addition, the authors observed a threshold acetylene coverage of 0.3 ML, below which benzene formation does not occur. They postulated that the reactant molecules are too far apart and steric crowding is necessary to form benzene in the low coverage regime. This postulate was supported by experiments with coadsorbed NO, a spectator molecule, which helped compress the 2D acetylene monolayer and increased the yield of benzene for the same surface coverage of acetylene. Furthermore, the authors provided evidence for the existence of a C_4_ intermediate along the pathway to benzene formation. Kyriakou *et al.* demonstrated that acetylene trimerizes to benzene on the Cu(111) surface but the reaction was not fully selective to benzene, as acetylene was also converted to ethylene, butadiene, and cyclooctatetraene.^37^ Using isotopically labeled experiments Kyriakou *et al.* demonstrated that the formation of butadiene requires C–H bond scission whereas formation of benzene occurs through a pure C–C coupling mechanism. The authors hypothesized that the C_4_ metallocycle is a key intermediate which can be hydrogenated to produce butadiene or coupled with an additional acetylene molecule to form benzene. Moreover, Baddeley *et al.* showed that PdAu bimetallic surfaces are active for acetylene cyclotrimerization to benzene, among other products. They demonstrated that PdAu surfaces are more active than pure Pd(111) and observed a dependence of the benzene yield on Pd coverage, theorizing two main factors dictated the effectiveness of the alloy system: rehybridization of the adsorbed acetylene and weaker adsorption of benzene on PdAu, which facilitates reaction.^35^

More recently, Boudjahem *et al.* reported that nanoparticle Ni/SiO_2_ catalysts perform acetylene cyclotrimerization to benzene in the presence of hydrogen.^38^ The authors found that the partial pressure of hydrogen and acetylene, the reaction temperature, and Ni loading all determine the conversion of acetylene and the product distribution. By lowering the Ni loading and reaction temperature, they were able to increase the benzene selectivity to a maximum of ∼52% in the presence of water vapor at conversions of ∼3%. Together, these studies provide an understanding of the mechanism behind acetylene cyclotrimerization to benzene and the critical role of the C_4_ intermediate. However, on extended metal surfaces like Cu and Pd, 100% selectivity towards benzene remains elusive due to their ability to break C–H bonds leading to by-products such as ethylene, ethane, and butane in addition to the cyclotrimerization to benzene pathway.

Given the Au-based findings described above, we examined acetylene cyclotrimerization to benzene on Ag(111) because it is generally inert like Au,^39^ but considerably cheaper. Furthermore, low index Ag surfaces do not reconstruct to large surface unit cells like the 22 × √3 herringbone reconstruction exhibited by Au(111), which makes Ag much simpler to accurately model with theory. Using a combination of UHV surface science experiments and density functional theory (DFT) based calculations we discovered that acetylene cyclotrimerizes to benzene with 100% selectivity when densely packed on the Ag(111) surface. Our temperature programmed desorption (TPD) results demonstrate a clear threshold of acetylene surface coverage (>1 ML) for the formation on benzene and reveal that additional acetylene layers promote the conversion to benzene. STM results show the progression of surface acetylene structures and features consistent with the formation of C_4_ intermediates as the acetylene surface coverage is raised. Our DFT calculations indicate that formation of the C_4_ intermediate is the crucial step in determining acetylene reaction *vs.* desorption. Furthermore, these DFT calculations reveal how multilayers facilitate C_4_ formation by strengthening interactions with the surface in the transition state more than the initial state, which lowers the C_4_ formation barrier. Together these results demonstrate the possibility of 100% selective benzene formation on Ag based catalysts and provide mechanistic insights for potential catalyst development.

## Methods

### Temperature-programmed desorption (TPD)

TPD studies were conducted using a UHV chamber with a base pressure < 1 × 10^−10^ mbar equipped with a Hiden Hal RC 201 mass spectrometer which could be advanced to ∼1 mm from the crystal face. The Ag(111) crystal could be cooled to cryogenic temperatures (∼85 K) using liquid nitrogen cooling and heated to 750 K *via* resistive heating of tungsten support wires. The Ag(111) crystal was cleaned using multiple Ar^+^ bombardment cycles (∼2 μA drain current, 1.5 kV) and annealing to 750 K. Acetylene (atomic absorption grade, Airgas) was exposed to the crystal at 90 K. TPDs were performed with a linear heating rate of 1.5 K s^−1^. Masses tracked: acetylene (*m*/*z* = 26), ethylene (*m*/*z* = 28), ethane (*m*/*z* = 30), 1,3-butadiene (*m*/*z* = 54), butene (*m*/*z* = 56), benzene (*m*/*z* = 78), and cyclooctatetraene (*m*/*z* = 104).

### Scanning tunneling microscopy (STM)

STM experiments were performed on a low-temperature scanning tunneling microscope (Infinity by Scienta Omicron) in UHV with a base pressure < 1 × 10^−11^ mbar in the scanning chamber and ∼1 × 10^−10^ mbar preparation chamber. The Ag(111) single crystal sample was cleaned with a series of Ar^+^ bombardment and 1000 K annealing cycles. Acetylene was exposed to the sample at ∼25 K, while STM imaging was performed at ∼13 K with a W tip.

### Computational methods

The DFT calculations were performed with the Vienna *Ab initio* Simulation Package (VASP).^40,41^ The Perdew–Burke–Ernzerhof generalized gradient approximation (GGA-PBE)^42^ exchange–correlation functional and the Tkatchenko–Scheffler method^43^ for van der Waals interactions were used in all the calculations. The cutoff energy was set to 400 eV. A 10 × 7 × 1, 7 × 7 × 1, or 3 × 5 × 1 *k*-point mesh was used for 2 × 3 × 4, 3 × 3 × 4, and 7 × 4 × 4 unit cells, respectively. The top two layers and adsorbates were fully relaxed during the optimization. The transition states were optimized by the dimer method^44^ and were confirmed by frequency calculations. The structures were visualized by VMD.^45^ For STM image simulations, the local density of states was sampled over an energy range corresponding to a bias voltage of −300 mV.

## Results and discussion

**Fig. 1:**
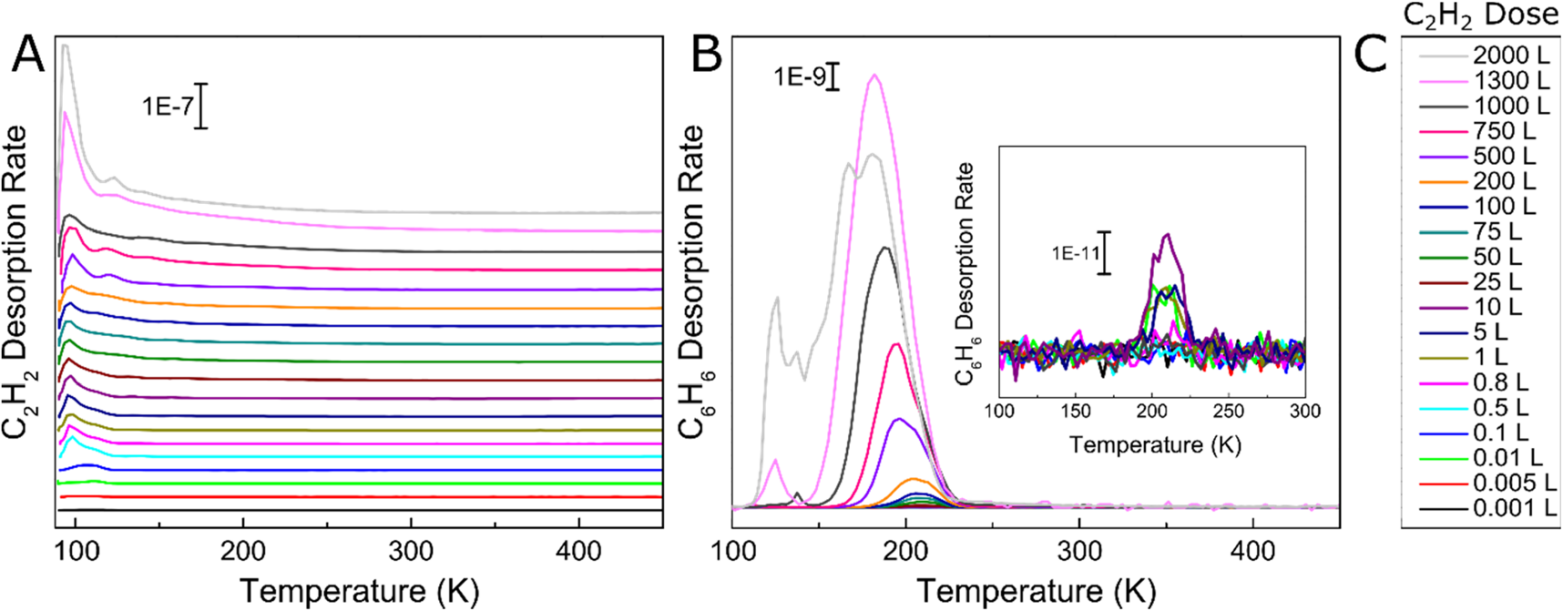
TPD traces of (A) increasing Langmuir doses of C_2_H_2_ on Ag(111) and (B) corresponding C_6_H_6_ production. (C) Legend for C_2_H_2_ and C_6_H_6_ traces. All C_2_H_2_ doses were performed at 90 K and the inset highlights benzene production from acetylene doses of 10 L and below.

We began by studying the effect of acetylene surface coverage on the selectivity and activity of the acetylene cyclotrimerization reaction on Ag(111) with UHV TPD. This technique enables determination of the reaction selectivity as well as the overall conversion of acetylene to benzene. We exposed the 90 K Ag(111) sample to various Langmuir (1 L = 1 × 10^−6^ torr s) doses of acetylene, ramped the temperature at 1.5 K s^−1^, and tracked the production of benzene with TPD as seen in Fig. 1. Fig. 1A shows acetylene desorption as a function of acetylene coverage and two distinct acetylene desorption features at ∼90 K and ∼130 K are seen. The lower temperature monolayer peak saturates at ∼10 L acetylene while the higher temperature desorption peak (attributed to acetylene stabilized by C_4_ intermediate formation, discussed later) keeps growing as the acetylene coverage increases beyond 10 L. The broad multilayer peak centered at ∼100 K continues to grow with increasing amounts of acetylene deposited, albeit at a slower rate. Importantly, very small amounts of benzene production occur below 1 L acetylene as seen in Fig. 1B, consistent with the surface acetylene coverage dependence observed in previous studies.^46,47^ The growth of the reactively formed benzene peak is also consistent with the previous benzene adsorption studies on the Ag(111) surface.^48^ However, to the best of our knowledge this is the first report of acetylene cyclotrimerization to benzene on any Ag surface. A previous study on Ag(110) concluded that adsorbed acetylene desorbs without reaction between 100 and 160 K,^49^ and another study on Ag(111) showed the reversible desorption of acetylene without trimerization.^50^ However, our results are still consistent given that both of these previous studies examined much lower acetylene surface coverages than the range studied herein. Furthermore, Ag atoms supported on MgO thin films have also been shown to be inactive for the cyclotrimerization of acetylene whereas Pd and Rh atoms show 100% selective acetylene to benzene conversion.^19^

To quantify the reaction selectivity using TPD, we tracked acetylene and benzene at *m*/*z* 26 and 78 and also *m*/*z* of 28, 30, 54, 56 and 104 for ethylene, ethane, 1,3 butadiene, butene and cyclooctatetraene, respectively, which were common side-products in previously reported acetylene trimerization studies.^31,37^ TPD traces corresponding to these products were all flat, with the exception of ethylene (Fig. S2†). However, the observed ethylene appears to come from the chamber background as opposed to being reactively formed on the surface (Fig. S3†). Specifically, experiments in which acetylene and fully deuterated benzene were deposited on Ag(111) and TPD was performed show that the ratio of C_6_D_6_ (84) to C_6_D_5_H (83) on the surface closely resembles the ratio observed during a background C_6_D_6_ dose revealing that no scrambling of hydrogen into the fully deuterated benzene occurs as seen in Fig. S3.† This result indicates that C–H bonds are not broken on the Ag(111) surface and therefore there is no supply of H needed to form ethylene on the Ag(111) surface. Further evidence for the observed ethylene originating from background adsorption comes from the fact that the Ag(111) surface is equally active for many reaction cycles without cleaning in between, indicating that there is no buildup of surface carbon, as would be expected if ethylene were a real product that required a supply of H from acetylene.

This demonstration that the acetylene trimerization to benzene is 100% selective on Ag(111) is supported by literature reports of the inability of the Ag(111) surface to break C–H bonds^51–54^ which is a requirement to form products like ethylene and butadiene.^17,21,29,36^ Furthermore, given that benzene is aromatic while cyclooctatetraene is not, benzene is the preferred product both enthalpically and entropically.^55,56^ Specifically, enthalpically, due to its aromatic ring, benzene is more thermodynamically stable product than cyclooctatetraene. Entropically the formation of a smaller gas phase molecule (benzene) *versus* cyclooctatetraene makes the formation of benzene thermodynamically more favorable.

**Fig. 2:**
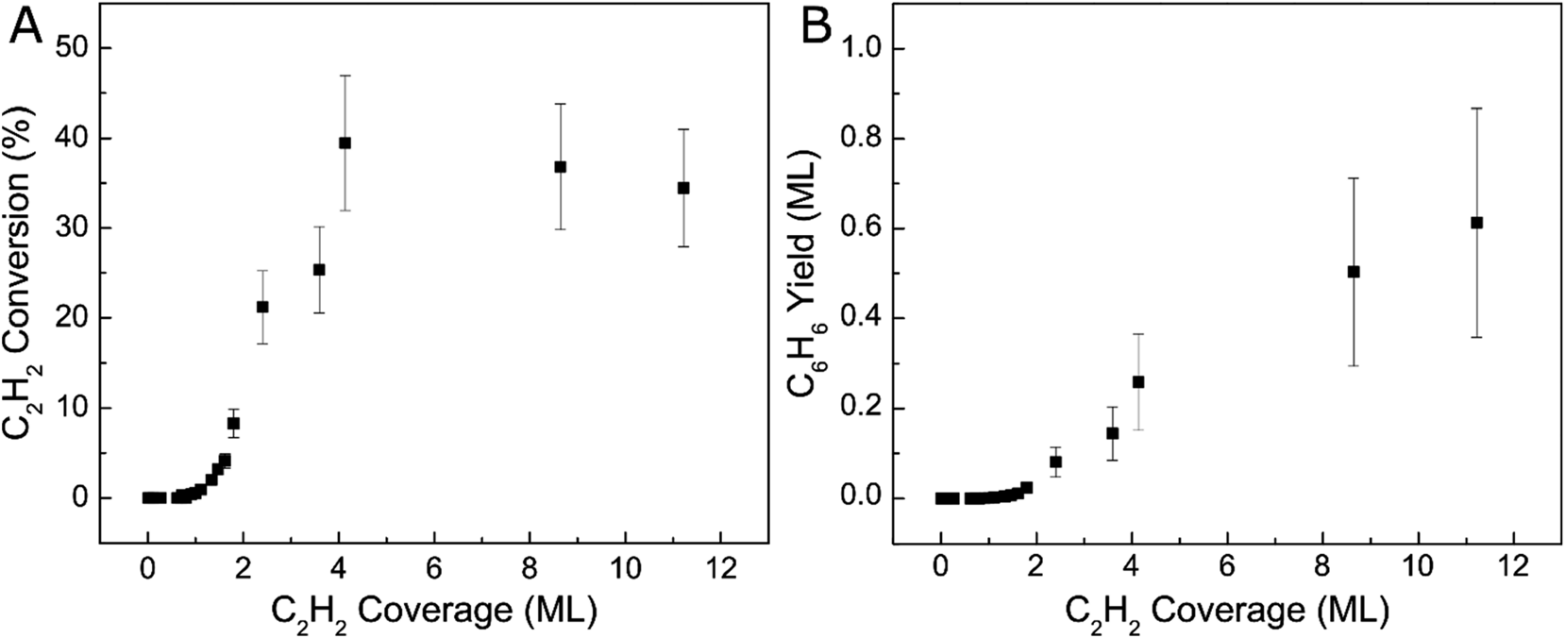
Coverage-dependent acetylene cyclotrimerization to benzene on Ag(111). (A) Acetylene conversion with respect to acetylene surface coverage and (B) benzene yield in monolayers plotted against acetylene surface coverage.

We further investigated the reactivity of Ag(111) towards acetylene cyclotrimerization by integrating the acetylene and benzene TPD peaks then applying correction factors to account for fragmentation pattern, ionization cross-section, and mass spectrometer quadrupole sensitivity.^57^ We then calculated the experimental conversion of acetylene as a ratio between the acetylene converted to benzene and the total amount of acetylene on the surface, as depicted in Fig. 2. The monolayer coverages of acetylene and benzene were defined by the saturation point for the low-temperature (90 K) acetylene peak and a separate uptake for benzene on Ag(111) respectively (see Fig. S2†). Fig. 2A demonstrates that acetylene conversion to benzene is very low when the acetylene surface coverage is low. However, the conversion increases abruptly when a saturated acetylene monolayer is formed and reaches a maximum of ∼40% at ∼4 ML. We hypothesize this phenomenon is due to the additional acetylene layers which facilitate tighter packing of acetylene molecules, leading to lower barriers for coupling, which will be explored further with DFT.^31^ Our TPD data indicate that additional acetylene adsorption beyond 4 ML still produces more benzene as shown in Fig. 2B, but does not improve the conversion. This may be due to the fact that acetylene does not grow strictly layer-by-layer on Ag(111), or due to effects beyond the second layer.^58^ Moreover, the total benzene yield continues to increase with acetylene coverage and does not saturate up to 12 ML acetylene. These results highlight the previously unconsidered role of 3D molecular overlayers on the conversion of acetylene to benzene.

**Fig. 3:**
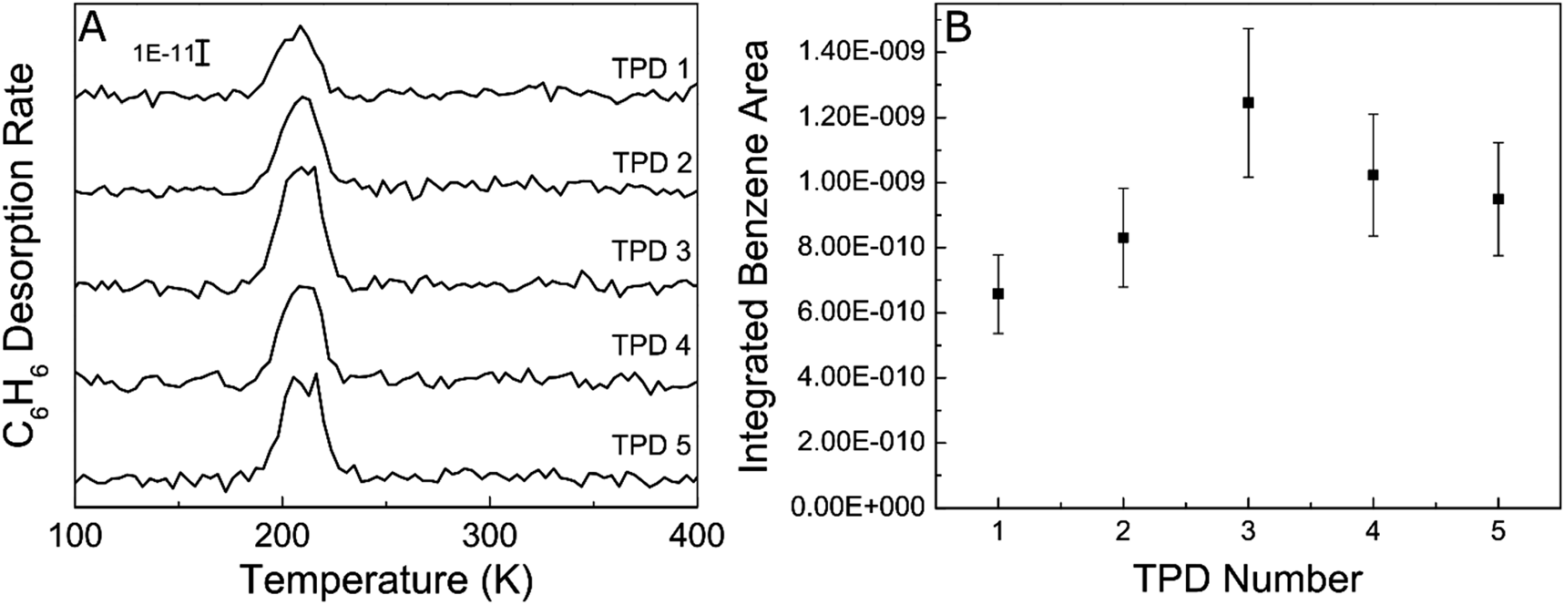
(A) Consecutive TPD experiments showing C_6_H_6_ yield after 10 L C_2_H_2_ exposure on Ag(111) at 90 K. (B) Benzene desorption peak areas calculated by integrating the benzene desorption peaks in panel (A). The error bars were calculated repeating 10 L acetylene exposures 5 times and taking the % error of the benzene yields.

In order to probe the robustness of Ag, we examined if the acetylene trimerization reaction poisoned the Ag(111) surface since catalyst deactivation was common in previously reported trimerization systems like Ni/SiO_2_, SiO_2_/Si(100) and fluorine on alumina.^17,38,59^ Specifically, we performed five consecutive cycles of TPD experiments without cleaning the Ag(111) in between as shown in Fig. 3. As is evident from the benzene desorption TPD results, the benzene yield in each TPD experiment is approximately the same, demonstrating that Ag(111) is not poisoned by the buildup of carbonaceous fragments like other active trimerization surfaces. This result is consistent with the inability of the Ag(111) surface to break C–H bonds which leads to carbon deposition.^60^ Lack of poisoning is also consistent with 100% selectivity because side products such as ethylene would lead to carbon build up and result in deactivation.

**Fig. 4:**
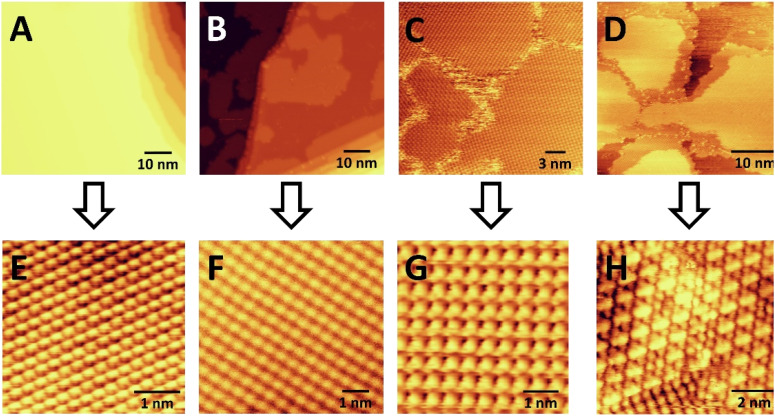
STM images of (A) pure Ag(111), (B) 1 L C_2_H_2_ on Ag(111), (C) 3 L C_2_H_2_ on Ag(111), (D) 10 L C_2_H_2_ on Ag(111), and (E) atomically resolved STM image of pure Ag(111). STM images in panels (F) and (G) show the square packing of C_2_H_2_ at lower acetylene coverage. Panel (H) shows the hexagonal packing of the C_2_H_2_ at higher coverage and the appearance of dumbbell shaped intermediates. C_2_H_2_ was exposed to the surface at ∼25 K and the images were taken at ∼13 K. Imaging conditions: (A) and (E) 0.2 nA and 600 mV, (B) 0.02 nA and 900 mV, (F) 0.03 nA and 900 mV, (C) 0.1 nA and −700 mV, (G) 0.1 nA and −500 mV, (D) and (H) 0.1 nA and 800 mV.

We then studied acetylene cyclotrimerization to benzene at the molecular level by conducting STM experiments. Fig. 4 panels (A) and (E) show large scale and atomically resolved images of the clean Ag(111) surface before C_2_H_2_ exposure. At low coverages (B + F) acetylene forms 2D islands of square-packed acetylene molecules on Ag(111) at 25 K. At monolayer coverage (which corresponds to packing density of 1 acetylene per 3.2 Ag surface atoms as calculated from STM experiments) (C) these islands merge into a complete monolayer composed of separate rotational domains of the square-packed acetylene structure due to their alignment with one of the three equivalent high-symmetry axes of the Ag(111) lattice. Four of these rotational domains can be seen in Fig. 4C and the inset in panel (G) shows the square packing structure of the acetylene monolayer. At acetylene coverages above one monolayer, growth of a second layer of acetylene is observed (D) which coincides with the appearance of dumbbell-shaped protrusions consistent with the proposed C_4_ intermediates within the first acetylene monolayer (H). Moreover, panel (H) reveals that the regions containing the intermediates have converted from a square to a hexagonal packing structure after the first monolayer is reached. These data indicate that the addition of >1 ML acetylene to Ag(111) causes a denser, hexagonal acetylene packing structure (1 acetylene per 2.7 Ag surface atoms) to form, which coincides with the appearance of dumbbell shaped protrusions which we speculate are the proposed C_4_ intermediates, and we investigate this point further with DFT.

In order to further examine the structure and stability of various configurations and coverages of acetylene, DFT was used to calculate energetics and generate simulated STM images. We first studied the acetylene adsorption energy in several structures and for different coverages, initially focusing on mimicking the square-packed structures seen in STM. The calculations generally predict stronger adsorption as the acetylene packing density increases, which is consistent with the experimental result that acetylene forms compact islands surrounded by bare Ag(111) at <1 ML coverage (see Fig. 4B). For example, the adsorption energy of a single, isolated acetylene is −0.29 eV, while the presence of a co-adsorbed, parallel acetylene in a 2 × 3 unit cell strengthens the adsorption energy to −0.35 eV (Fig. S5a†). This strengthening is likely due to van der Waals interactions between acetylene molecules and explains the islanding observed in experiments (Fig. 4B).

To mimic the structure seen in experimental STM at higher acetylene coverage containing the proposed C_4_ intermediates, the simulated STM image of 3 C_2_H_2_ molecules and 2 C_4_H_4_ intermediates in a 7 × 4 unit cell is shown in Fig. S5.† This simulated STM image is similar to the features seen in Fig. 4H, indicating that the large bright species in Fig. 4H are likely the C_4_ intermediates surrounded by acetylene molecules which appear as smaller protrusions in the simulation.

**Fig. 5:**
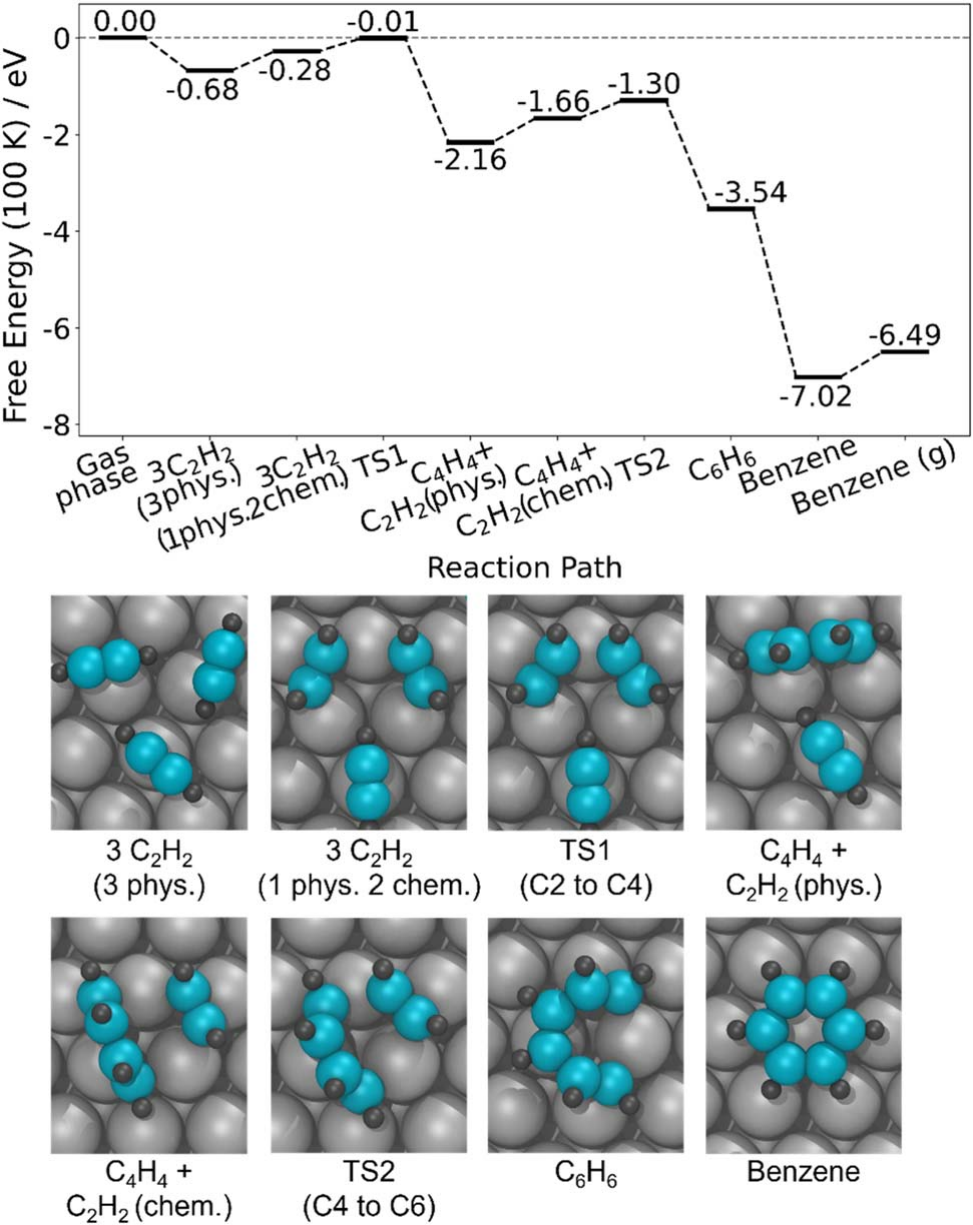
Calculated reaction pathway of acetylene trimerization to benzene on Ag(111). The optimized structures along the pathway in a 3 × 3 unit cell are shown. Free energies calculated at 100 K, 1 atm.

To understand critical steps in the reaction, we studied the full acetylene trimerization pathway to benzene on a 3 × 3 surface cell (Fig. 5). At 100 K and 1 atm, the transition state for forming the C_4_ intermediate is very close to zero in free energy (−0.01 eV), where the zero is set to the acetylene molecules in the gas phase. Therefore, the competition between reaching this transition state and acetylene desorption will control the reaction efficiency. Even small changes in the energy of this transition state could move it above or below the energy zero and therefore have a large impact on the conversion to benzene *vs.* desorption. Furthermore, Fig. 5 illustrates that once the C_4_ intermediate is formed, the rest of the reaction pathway to benzene is significantly below 0 eV, generally downhill in energy, and features no large barriers, further indicating that the formation of the C_4_ intermediate has the largest degree of rate control.^61^ These DFT calculations also indicate that in order to form the C_4_ intermediate, two physiosorbed acetylene molecules must first become chemisorbed and bind to one Ag atom. For a single acetylene molecule, the chemisorbed state is 0.09 eV less stable than the physisorbed state, and this difference is expected to depend on the structure and packing density of the acetylene monolayer. Therefore, understanding the energetics of moving from the all-physisorbed state to a state with two chemisorbed acetylene molecules and then to the transition state for C_4_H_4_ formation as a function of acetylene coverage and structure should provide deeper insight into the reaction pathway and ultimate conversion to benzene.

**Fig. 6:**
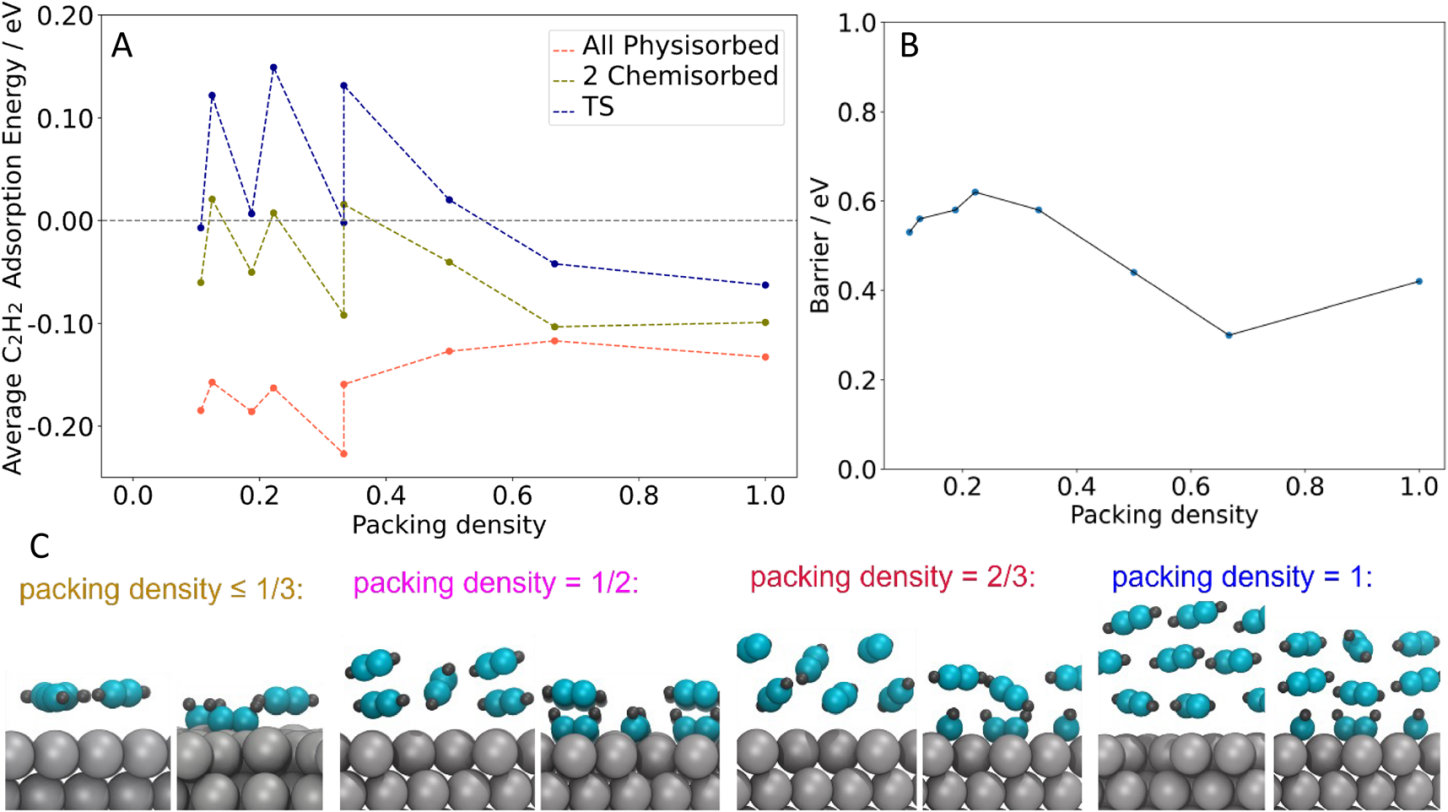
The C_4_ intermediate formation step with different packing densities. (A) Free energies for C_2_H_2_ in various states on the Ag(111) surface as a function of packing density. (B) C_4_ formation barrier, calculated from the lowest energy state to the transition state (TS1), as a function of packing density. All packing densities given in this work are in terms of C_2_ unit per surface Ag atom. (C) Side views of DFT optimized structures of C_2_H_2_ adsorption on the Ag(111) surface with different packing densities.

We therefore studied the C_4_ formation step with DFT for several different acetylene packing structures in order to understand the influence of acetylene packing density and structure on benzene formation. Structures where all acetylene molecules are directly interacting with the surface were formed by placing two and three acetylene molecules in different unit cells and keeping the packing density ≤ 1 acetylene per 3 surface Ag atoms (for example, Fig. S5a†). Of these structures, three acetylene molecules in a 3 × 3 unit cell give the most stable configurations for both the all-physisorbed state and the state with two chemisorbed acetylene molecules. When increasing the packing density (see the structures in Fig. 6), our DFT calculations predict that acetylene molecules start forming the second layer when the packing density reaches 1 acetylene per 3 surface Ag atoms and there are three acetylene layers when the packing density is 1 : 1.

To examine the mechanism through which physisorbed multilayers may promote the formation of benzene, we calculated the average acetylene adsorption energy for the all-physisorbed structure (initial state, lowest energy), the state with two chemisorbed acetylene molecules (precursor to the transition state for C–C coupling), and the transition state for C_4_ formation (Fig. 6A). This allowed us to understand how the absolute and relative stabilities of these states change as a function of coverage. We also calculated the barrier for C_4_ formation, defined as the difference in energy between the transition state and the most-stable state, which is always the all-physisorbed state (Fig. 6B).

For the coverages where acetylene forms a single layer (packing density ≤ 1 acetylene per 3 surface Ag atoms), the energies of all three states are highly correlated, showing very similar shifts (see Fig. 6A). These shifts are primarily due to the number of acetylene molecules in the unit cell: the structures in question all have two or three acetylene molecules present, and when there are three present the energy is shifted down. However, these shifts have only a small effect on the barrier (see Fig. 6B).

On the other hand, when >1 ML of acetylene is present (packing density ≥ 1 acetylene per 3 surface Ag atoms, all in 2 × 3 unit cells), the average stability in the physisorbed state shifts in the *opposite* direction of the chemisorbed state and transition state (see Fig. 6A). The stabilization of the chemisorbed state at higher coverages is likely due to the second layer of acetylene moving closer to the surface during the chemisorption process, strengthening its interaction with the surface (see Fig. 6C). Examining the stability of just the physisorbed molecules in each state supports this picture (see Fig. S7†). This trend applies to the transition state as well, resulting in a significant decrease in the barrier (Fig. 6B). The lowest barrier is 0.3 eV, from 4 acetylene molecules in a 2 × 3 unit cell (2 acetylene layers, packing density = 2 acetylene per 3 surface Ag atoms corresponding to ∼2 ML in experiment) Together, these results indicate that when multi-layers of acetylene are present, there is a driving force towards chemisorption: the increased energy for the chemisorbed or reacting molecules themselves is partially compensated by a stronger interaction between the second layer of acetylene and the Ag surface. This explains the effect of acetylene multilayers on the reaction, and also explains the DFT results which demonstrate how the reaction barriers decrease as the acetylene packing density increases beyond 1 acetylene per 3 surface Ag atoms, while the barriers are not sensitive to acetylene coverage at lower coverages than this. Additionally, the predicted trend is consistent with the experimental finding that benzene production begins around 1 ML acetylene, which corresponds to a packing density of roughly 1 acetylene per 3 surface Ag atoms (the square packing structure observed in STM contained 1 acetylene per 3.2 surface Ag atoms *vs.* the hexagonal packing structure which has 1 acetylene per 2.7 surface Ag atoms). Together, these results highlight the importance of the change in packing structure to a higher density, as occurs when the acetylene monolayer transitions from square to hexagonal, offering a lower reaction barrier that also benefits from multilayer acetylene adsorption.

Our results show a clear trend of a generally decreasing energetic barrier with increasing packing density, with quite significant variations in the barrier height. However, due to the complexity of the multilayer structures, we expect some quantitative error in the barrier heights due to the structures we use, in addition to the intrinsic error from DFT. Indeed, even our lowest barrier would suggest very slow formation of the C_4_ intermediates at 25 K, and yet STM suggests that these intermediates are formed at low temperature. In experiment, C_4_ intermediates may form at locations where the local molecular packing structure leads to particularly low reaction barriers.

To explain the high selectivity and lack of poisoning observed experimentally, we also calculated the possibility of C–H bond cleavage in acetylene on Ag(111). As shown in Fig. S8,† the transition state and product of C–H cleavage have much higher energies than those in the trimerization pathway. In particular, desorption (0.29 eV) is far more favorable than C–H bond cleavage (barrier of 1.43 eV). This implies that C–H bonds in acetylene are unlikely to be broken on Ag(111) and explains the experimental findings that acetylene can be transformed to benzene with 100% selectivity on Ag(111) *versus* more reactive surfaces like Pd(111) and PdAu alloys which are capable of C–H activation and are thus considerably less selective to benzene.

## Conclusions

Using temperature programmed desorption, molecular scale imaging, and DFT, we discovered that acetylene cyclotrimerizes to benzene with 100% selectivity on the Ag(111) surface. Specifically, benzene formation requires a critical surface coverage of acetylene > 1 ML, after which the benzene formation dramatically increases. Importantly, the Ag(111) surface does not deactivate after multiple reaction cycles exhibiting resilience to poisoning unlike the Cu, Ni, Si and Pd based cyclotrimerization systems reported in the literature. STM imaging demonstrated that above the critical coverage of acetylene, the acetylene monolayer converts from a square to a denser hexagonal packing structure, and bright features consistent with the proposed C_4_ intermediates are observed. DFT results indicate that reaching the transition state for forming the C_4_ intermediate is most important for determining the reaction efficiency and that this transition state, as well as the transformation of physisorbed acetylene to chemisorbed acetylene, can be promoted by the adsorption of additional acetylene layers. We attribute the lack of any side products such as ethylene and butadiene to the inability of the Ag(111) surface to perform dehydrogenation, which has a much higher calculated energy barrier. Together, these results provide fundamental understanding of the 100% selective acetylene cyclotrimerization to benzene on Ag(111) which we hope informs the design of Ag-based nanoparticle catalysts for this reaction which will be of greater importance as the move away from heavy hydrocarbon feedstocks continues.

## Data availability

The datasets supporting this article have been uploaded as part of the ESI.†

## Author contributions

Volkan Çınar carried out the TPD experiments, data analysis and writing of the original manuscript. Elizabeth E. Happel performed the STM experiments. Nipun T. S. K. Dewage conducted control experiments. E. Charles H. Sykes supervised the TPD and STM experiments, data curation and writing of the original draft. Shengjie Zhang carried out the DFT calculations under Matthew M. Montemore's supervision who also edited the manuscript.

## Conflicts of interest

There are no conflicts of interest to declare.

## Supplementary Material

SC-015-D4SC01053A-s001
