# Supplementary material for: 100% selective cyclotrimerization of acetylene to benzene on Ag(111)
Source: Chem Sci. 2024 Apr 10;15(18):6716–25. doi: 10.1039/d4sc01053a (PMC11077525; doi:10.1039/d4sc01053a)
Supplement: SC-015-D4SC01053A-s001 [file SC-015-D4SC01053A-s001.pdf]

Electronic Supplementary Material (ESI) for Chemical Science.
This journal is © The Royal Society of Chemistry 2024

100% Selective Cyclotrimerization of Acetylene to Benzene on Ag(111)

Volkan Çınar,[a] Shengjie Zhang,[b] Elizabeth E. Happel,[a] Nipun T. S. K. Dewage,[a] Matthew M. Montemore[*b], E. Charles H. Sykes[*a]

[a]*Department of Chemistry, Tufts University, Medford, Massachusetts 02155, United States; Email: charles.sykes@tufts.edu*

[b]*Department of Chemical and Biomolecular Engineering, Tulane University, New Orleans, Louisiana 70118, United States; orcid.org/0000-0002- 4157-1745; Email: mmontemore@tulane.edu*

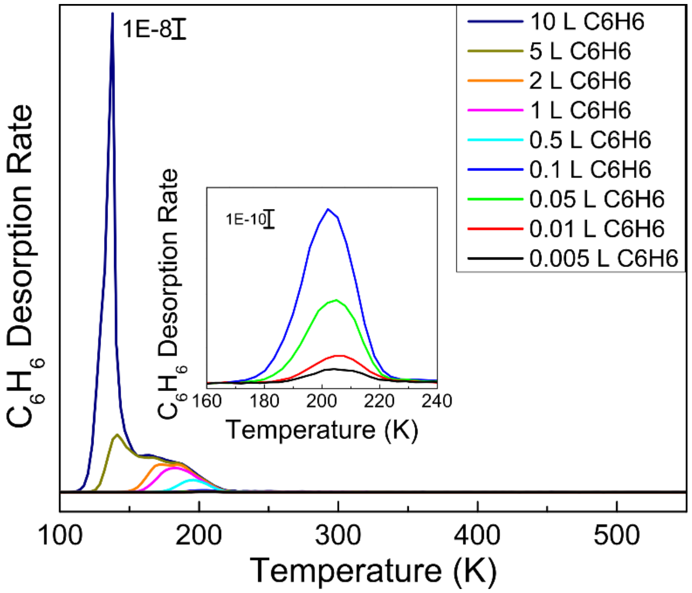

Fig. S1 TPD data of benzene (m/z = 78) uptake on Ag(111). Benzene was dosed at 90 K for each TPD experiment.

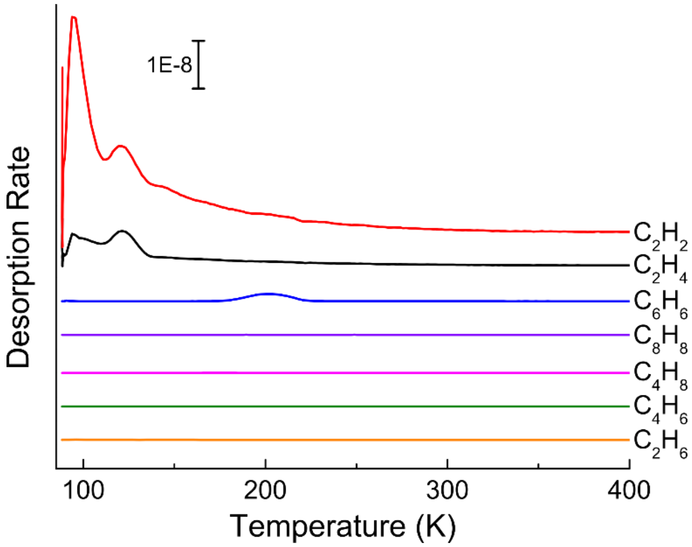

Fig. S2 TPD traces for acetylene (m/z = 26), ethylene (m/z = 28), ethane (m/z = 30), 1,3-butadiene (m/z = 54), butene (m/z = 56), benzene (m/z = 78), and cyclooctatetraene (m/z = 104). In this experiment 100 L of acetylene was deposited on Ag(111) at 90 K.

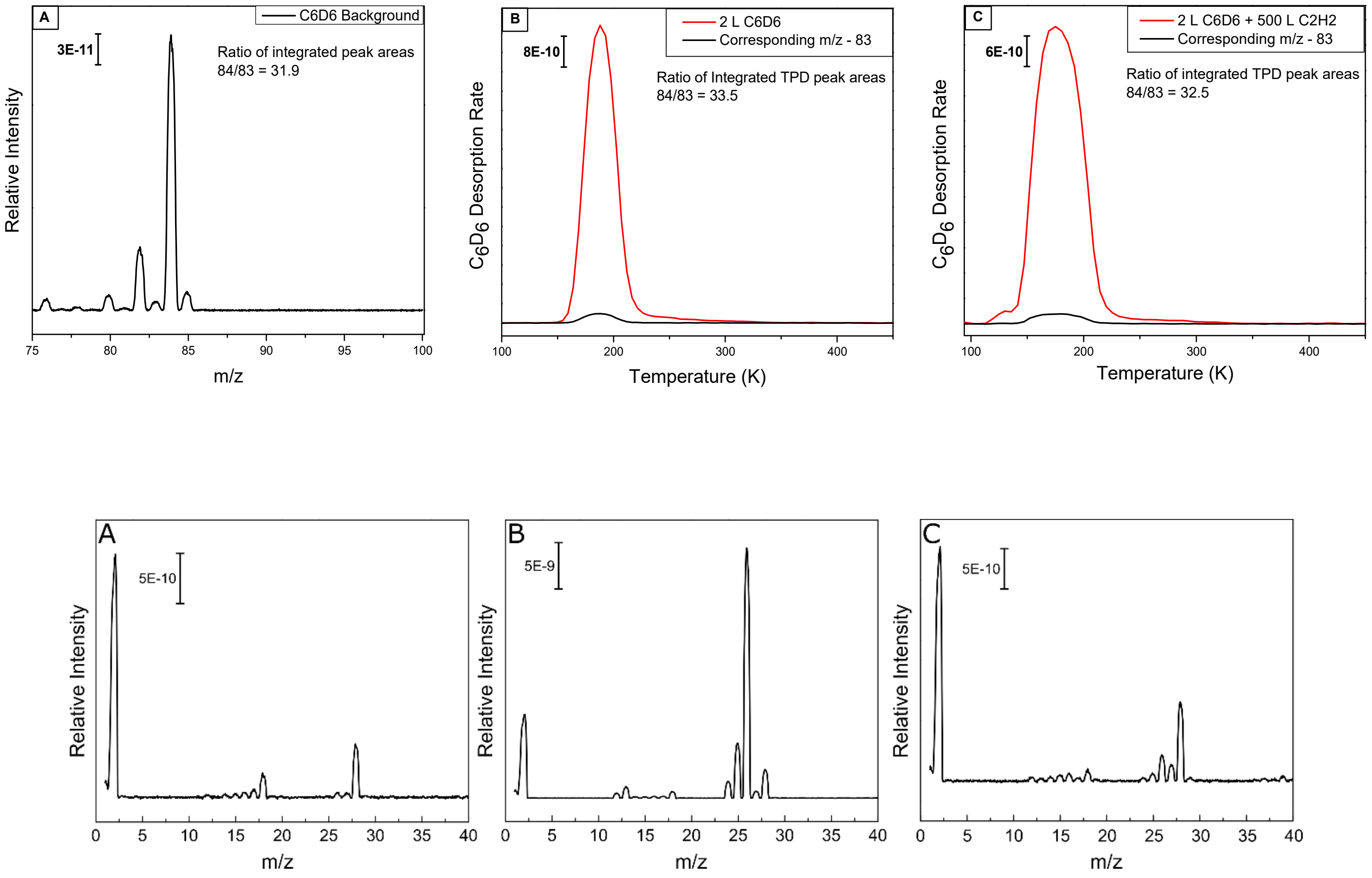

Figure S3: **UPPER** (A) Mass spectra for C_6D_6 and the ratio of C_6D_6 (m/z = 84) to C_6D_5H (m/z = 83) in the 5×10^{-9} mbar C_6D_6 background dose. (B) TPD data for C_6D_6 desorption from Ag(111) shows the ratio of C_6D_6 to C_6D_5H adsorbed on Ag(111) after a 2 L C_6D_6 exposure (C) TPD data for co-adsorbed C_6D_6 and C_2H_2 on Ag(111) illustrates the same C_6D_6 (m/z = 84) to C_6D_5H (m/z = 83) ratio, indicating no isotope scrambling between C_2H_2 and fully deuterated benzene occurred. **LOWER** Mass spectra for (A) UHV chamber background before 100 L acetylene dose showing prominent peaks at m/z 18 and 28 for water and CO, (B) 1×10^{-6} mbar of acetylene showing a prominent peak at m/z 26 and (C) UHV chamber background after 100 L acetylene dose showing a diminished m/z 26 signal but enhanced m/z 28 consistent with acetylene hydrogenation to ethylene on the UHV chamber walls. Background spectrum collected in panel A before was subtracted from the spectrum in panel B to distinctly show the changes induced by the introduction of acetylene.

1. Explanation of Ethylene Peak

In order to probe the origin of the ethylene peak in Fig. S2 and support the claim of 100% selectivity of acetylene trimerization we used mass spectrometry[1] to study isotope scrambling of the reactant and product molecules. Specifically, if ethylene is to be reactively formed on the Ag(111) surface, C-H bonds in acetylene must be broken to supply H. We therefore deposited C_6D_6 and regular acetylene on Ag(111) and monitored H/D exchange between molecules that would indicate C-H activation. Fig. S3A UPPER shows a mass spectrum recorded when 5×10^{-9} mbar of

C_6D_6 was introduced to the UHV chamber showing that the ratio of C_6D_6 (m/z = 84) to C_6D_5H (m/z = 83) in the incoming gas was 31.9. Similarly, after a 2 L C_6D_6 dose on Ag(111), the desorbing molecules in TPD have a C_6D_6 (m/z = 84) to C_6D_5H (m/z = 83) ratio 33.5 as shown in Fig. S3B UPPER. Then, TPD of co-adsorbed C_6D_6 and C_2H_2 on Ag(111) shown in Fig. S3C UPPER illustrates that the same C_6D_6 (m/z = 84) to C_6D_5H (m/z = 83) ratio (32.5) is observed, indicating no isotope scrambling occurs between C_2H_2 and fully deuterated benzene, and hence C-H bonds are not broken.

Furthermore, the expected ratio of m/z 26 to 28 according to the NIST[1] database is 999.9. However, as seen in Fig. S3 LOWER, during the acetylene dose the ratio of m/z 26 to 28 was 8.74. This indicates that acetylene (m/z 26) must be hydrogenating on the chamber walls to produce ethylene (m/z 28). Furthermore, after pump down to UHV the ratio of m/z 26 to 28 is 0.21 indicating that a significant amount of ethylene remains in the chamber background after the introduction of acetylene. This result indicates that the observed desorption of ethylene at low temperatures (around 95 and 120 K) in the cyclotrimerization experiments originates from background ethylene adsorption.

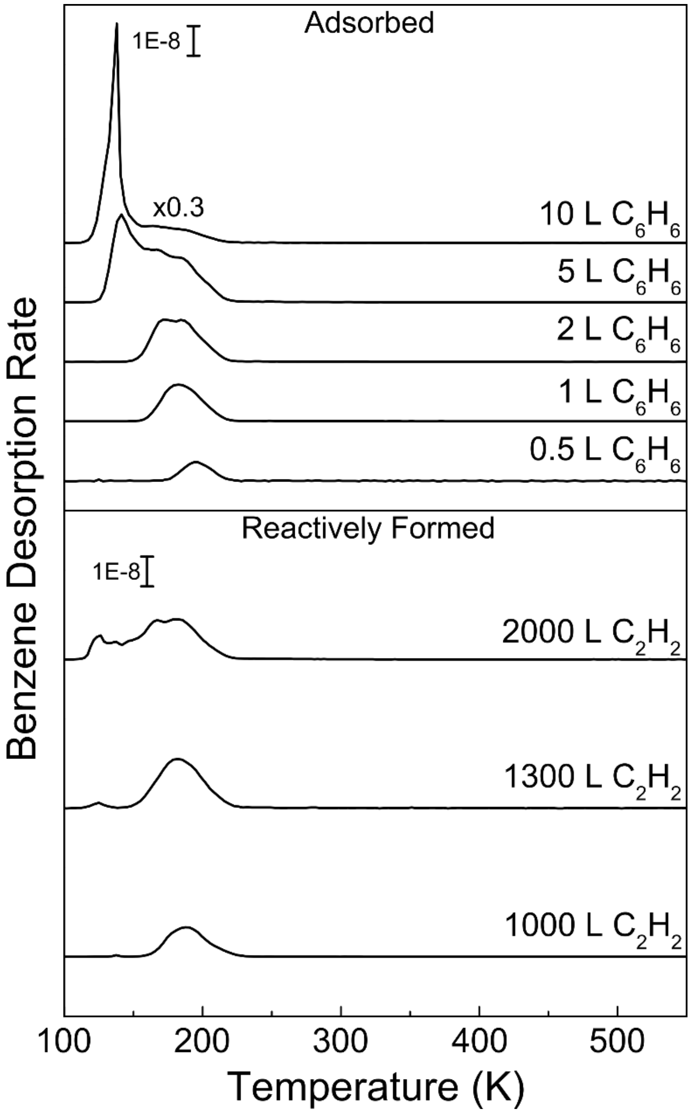

Fig. S4 Benzene desorption rates from adsorbed benzene (top) and acetylene trimerization (bottom) on Ag(111).

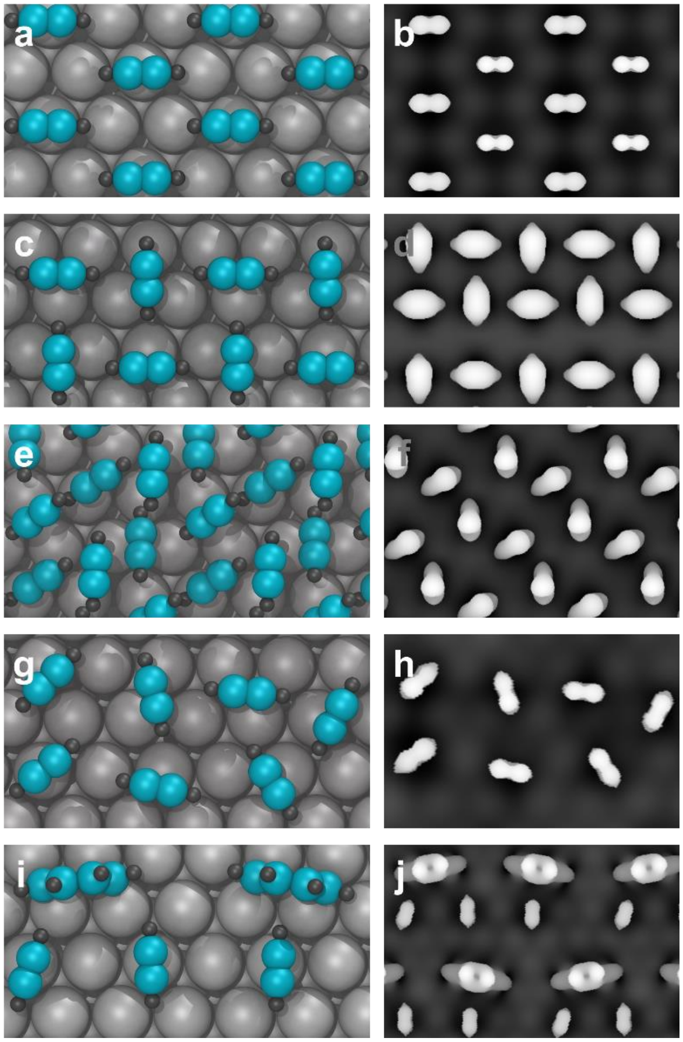

Fig. S5 DFT optimized structures and simulated STM images. (a), (b): 2 C_2H_2 molecules in a 2x3 unit cell (packing density = 1/3), mimicking the square packing structure. (c), (d): 4 C_2H_2 molecules in a 4x3 unit cell (packing density = 1/3), mimicking the square packing structure. (e), (f): 4 C_2H_2 molecules in a 2x3 unit cell (packing density = 2/3). (g), (h): 7 C_2H_2 molecules in a 7x4 unit cell (packing density = 1/4). (i), (j): 3 C_2H_2 molecules and 2 C_4H_4 groups in a 7x4 unit cell.

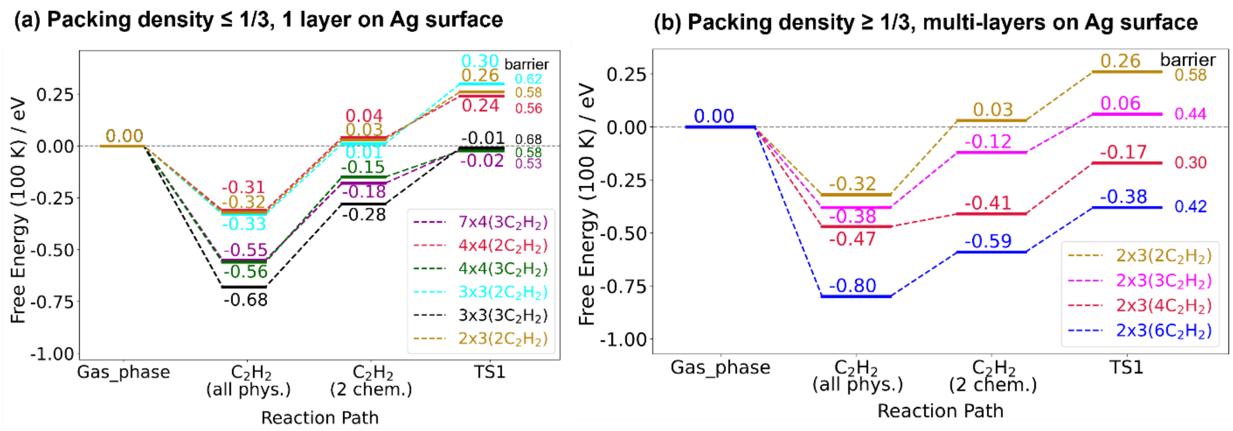

Fig. S6 The C_4 formation step with different packing densities. Barriers are calculated from the lowest energy state to the transition state (TS1). (a) Free energies for a single C_2H_2 layer with various structures on a Ag(111) surface, all with packing density ≤ 1/3. (b) Free energies for different amounts of C_2H_2 in a 2x3 unit cell, forming multi-layers with packing density ≥ 1/3. Note that energies cannot be compared across different numbers of acetylene molecules per unit cell.

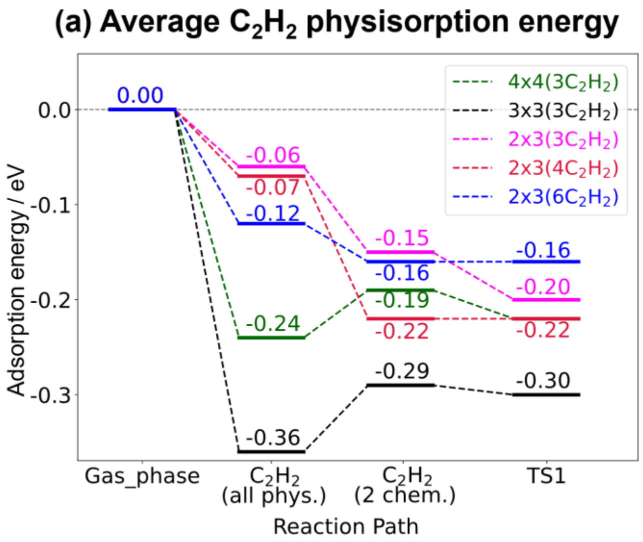

Fig. S7 Average adsorption energy of physisorbed C_2H_2 molecules (i.e., not including any chemisorbed or reacting molecules). As the packing density increases, the physisorbed molecules adsorb more strongly in the two-chemisorbed state and the transition state as compared to the all-physisorbed state. This counteracts the increase in energy for the chemisorbed or reacting molecules and facilitates reaction.

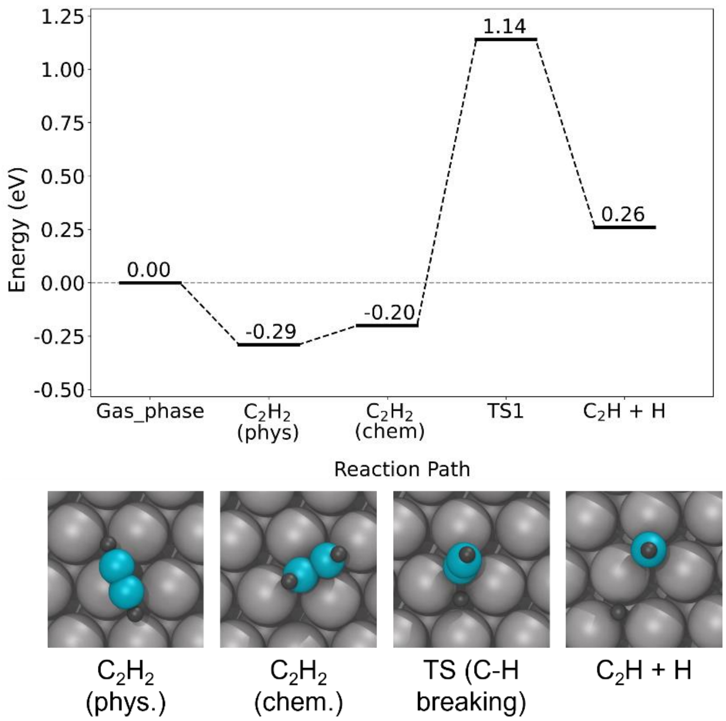

Fig. S8 Acetylene C-H bond-breaking pathway on the Ag(111) surface. C-H bond breaking is both kinetically and thermodynamically unfavorable.

References

(1) NIST Chemistry WebBook https://webbook.nist.gov/
